# Adolescents’ responses to the promotion and flavouring of e-cigarettes

**DOI:** 10.1007/s00038-015-0769-5

**Published:** 2015-12-09

**Authors:** Allison Ford, Anne Marie MacKintosh, Linda Bauld, Crawford Moodie, Gerard Hastings

**Affiliations:** Centre for Tobacco Control Research, Institute for Social Marketing, University of Stirling, Stirling, UK; UK Centre for Tobacco and Alcohol Studies, University of Stirling, Stirling, UK

**Keywords:** E-cigarettes, E-cigarette use, Adolescents, Marketing, Promotion, Flavours

## Abstract

**Objectives:**

The purpose of the study is to examine adolescents’ awareness of e-cigarette marketing and investigate the impact of e-cigarette flavour descriptors on perceptions of product harm and user image.

**Methods:**

Data come from the 2014 Youth Tobacco Policy Survey, a cross-sectional in-home survey conducted with 11–16 year olds across the UK (*n* = 1205). Adolescents’ awareness of e-cigarette promotion, brands, and flavours was assessed. Perceptions of product harm, and likely user of four examples of e-cigarette flavours was also examined.

**Results:**

Some participants had tried e-cigarettes (12 %) but regular use was low (2 %) and confined to adolescents who had also smoked tobacco. Most were aware of at least one promotional channel (82 %) and that e-cigarettes came in different flavours (69 %). Brand awareness was low. E-cigarettes were perceived as harmful (*M* = 3.54, SD = 1.19) but this was moderated by product flavours. Fruit and sweet flavours were perceived as more likely to be tried by young never smokers than adult smokers trying to quit (*p* < 0.001).

**Conclusions:**

There is a need to monitor the impact of future market and regulatory change on youth uptake and perceptions of e-cigarettes.

## Introduction

It is well established that exposure to, and appreciation of, tobacco marketing is linked with youth smoking and smoking susceptibility (Lovato et al. 2011; National Cancer Institute 2008). Similarly, evidence shows that tobacco flavourings appeal to young and novice smokers, particularly fruit, candy, and alcohol flavours (Carpenter et al. 2005; Wayne and Connolly 2002). In recent years, electronic cigarettes (e-cigarettes) have been marketed as an alternative to smoking. These devices do not contain tobacco, and national regulatory bodies in some countries, including the UK, have made it clear that they are less harmful than tobacco (MHRA 2013; NICE 2013). However, some e-cigarettes closely resemble cigarettes and the marketing strategies used to promote them have been similar to those used for tobacco (de Andrade et al. 2013). Research exploring how young people respond to e-cigarette marketing and flavours has been lacking.

A review of the literature exploring the impact of e-cigarettes on children found that, whilst there is evidence of increased youth exposure to advertisements (Duke et al. 2014), the effects of e-cigarette marketing and the availability of flavoured e-liquids on youth use are unknown (Durmowicz 2014). There is some evidence that adult exposure to e-cigarette advertising can increase interest in trying the product (Pepper et al. 2014), affect perceptions of product-related harms (Tan et al. 2015), and is associated with use (Harrington et al. 2014). Observing vaping in e-cigarette advertisements is also linked with an increase in daily adult smokers’ urge to smoke (Maloney and Cappella 2015). Furthermore, positive appraisal of advertisements has been associated with intended use among college students (Trumbo and Kim 2015).

Advertising is of course just one type of promotion. E-cigarette promotion takes place in multiple channels, including those previously used for traditional cigarettes and for other consumer products, such as product and packaging design, point-of-sale, billboards, radio and TV advertising, sponsorship, traditional print media, and celebrity endorsements, plus an array of online and social media options (de Andrade et al. 2013). This has been supported by increasing promotional expenditures (Kornfield et al. 2014) and resulted in concern about possible targeting of young people. Content analyses of e-cigarette promotions have found that it often conveys messages of sociability and sexuality (Richardson et al. 2014), which may tap into adolescent concerns about group and gender identity (Amos and Bostock 2007). It is clear, therefore, that research exploring the effects, if any, of e-cigarette promotion on children is important.

In addition to the potential influence e-cigarette promotions may have on youth, there is a need to understand the role that aspects of product design, such as flavourings, have on how children perceive e-cigarettes, particularly as it has been suggested that e-cigarette flavours could appeal to young non-smokers (Giovenco et al. 2014; Hughes et al. 2015). What is known is that there are myriad e-cigarette flavours available. An analysis of brands advertised and sold on the internet found that in a 17-month period, between 2012 and 2014, 242 new flavours were introduced each month. By January 2014, the total number of flavour offerings exceeded 7700 (Zhu et al. 2014).

This growth in flavours may not be surprising, as products have diversified and adult smokers who use e-cigarettes express preferences for a range of flavours. The most recent Eurobarometer survey found that amongst current adult users of e-cigarettes, flavour was the most important factor in their choice of product (39 % of respondents) followed by price (38 %) and nicotine content (27 %) (European Commission 2015). Smokers who successfully quit with e-cigarettes cite alternative flavours (other than tobacco) as important in breaking the link with smoking (Farsalinos et al. 2013). Smoking significantly increases the risk of impaired olfactory function (Vennemann et al. 2008). Smokers who quit report regaining their sense of taste and smell as one of the benefits of cessation, allowing greater appreciation of flavours. A choice of flavours may play a valuable role in e-cigarette product appeal to smokers who are trying to quit.

However, much as with promotion, few studies have explored young people’s perceptions of e-cigarette flavours. While two studies found little interest in flavoured e-cigarettes among teenagers (Pepper et al. 2013; Shiffman et al. 2015), another suggested that flavours encourage e-cigarette experimentation (Kong et al. 2014). A further two studies highlighted adolescent preference for sweet flavours (Krishnan-Sarin et al. 2015; McNeill et al. 2015). For example, a UK survey found that just over 50 % of 11- to 18-year-old ever-users reported that their last e-cigarette had contained a fruit flavour, but less than 10 % said it had contained a tobacco flavour (McNeill et al. 2015).

This study helps fill gaps in the literature. It examines awareness and use of e-cigarettes among UK adolescents, as well as awareness of e-cigarette promotion, branding, and flavours. It also investigates whether e-cigarette flavours affect perceptions of product harm and user image.

## Methods

### Design

Data came from Wave 7 of the Youth Tobacco Policy Survey (YTPS), a long-running, repeat cross-sectional study examining the impact of tobacco policies on adolescents. FACTS international, a market research company, recruited participants and conducted the fieldwork in August and September 2014. Parental and participant informed consent was obtained prior to each interview. The survey comprised an in-home face-to-face interview, followed by a self-completion questionnaire to gather more sensitive information. To maximise privacy, should anyone else be present where the interview was taking place, questions were displayed on showcards to enable participants to read responses from the card and give the number corresponding to their answer. Ethical approval was obtained from the Stirling Management School ethics committee.

### Sampling strategy

Using random location quota sampling, a sample of 11–16 year olds was drawn from households across the UK. Sampling involved random selection of 92 electoral wards stratified by Government Office Region and A Classification Of Residential Neighbourhoods (ACORN) classification (a geodemographic classification system that describes demographic and lifestyle profiles of small geographic areas) to ensure coverage of a range of geographic areas and socio-demographic backgrounds. Wards covering the islands, areas north of the Caledonian Canal, or those with fewer than three urban/suburban Enumeration Districts were excluded from the sampling frame for cost and practicality reasons. In each selected ward interviewers approach households until a quota of 15 interviews is obtained, balanced across gender and age. A total sample of 1205 was achieved. Comparative census data for England and Wales indicate that the sample was in line with national figures for gender and age (ONS 2012) and also in line with smoking prevalence among 11–15 year olds in England (Fuller 2015).

### Development of the survey items and testing

Research between April and July 2014 informed the development and refinement of the e-cigarette measures. Initially, six focus groups were conducted with 11–16 year olds to explore their knowledge of e-cigarettes, how they think about and respond to them, and the language and meanings they attach to them. A draft questionnaire was developed from the emerging themes using, as far as possible, the terms the young people used. This was piloted with 11 participants aged 11–16 years. Two professional market research interviewers were involved in administering the pilot questionnaire. Each interview was observed by a member of the research team, to test the flow of the questionnaire, timing, and comprehension of questions and visual stimuli. On completion of the questionnaire, the interviewer left the room and the researcher conducted an in-depth cognitive interview to assess participant understanding of the measures, relevance of questions and ability to respond.

### Measures

#### General information

Information was obtained on age and gender. Social grade was based on the UK demographic classifications system derived from the National Readership Survey and determined by the occupation of the chief income earner in the household. Never smokers were categorised as those who had ‘never tried smoking, not even a puff or two’. Ever smokers included those who indicated being regular smokers (at least one cigarette a week), occasional smokers (less than one a week), those who used to smoke and those who had tried smoking only once.

#### Awareness of e-cigarettes

Questions on e-cigarettes were introduced with: ‘Now we’d like you to think about electronic cigarettes, sometimes called e-cigarettes or e-shisha. E-cigarettes puff a vapour that looks like smoke but, unlike normal cigarettes, you don’t light them with a flame and they don’t burn tobacco. Have you ever heard of e-cigarettes?’

**Fig. 1:**
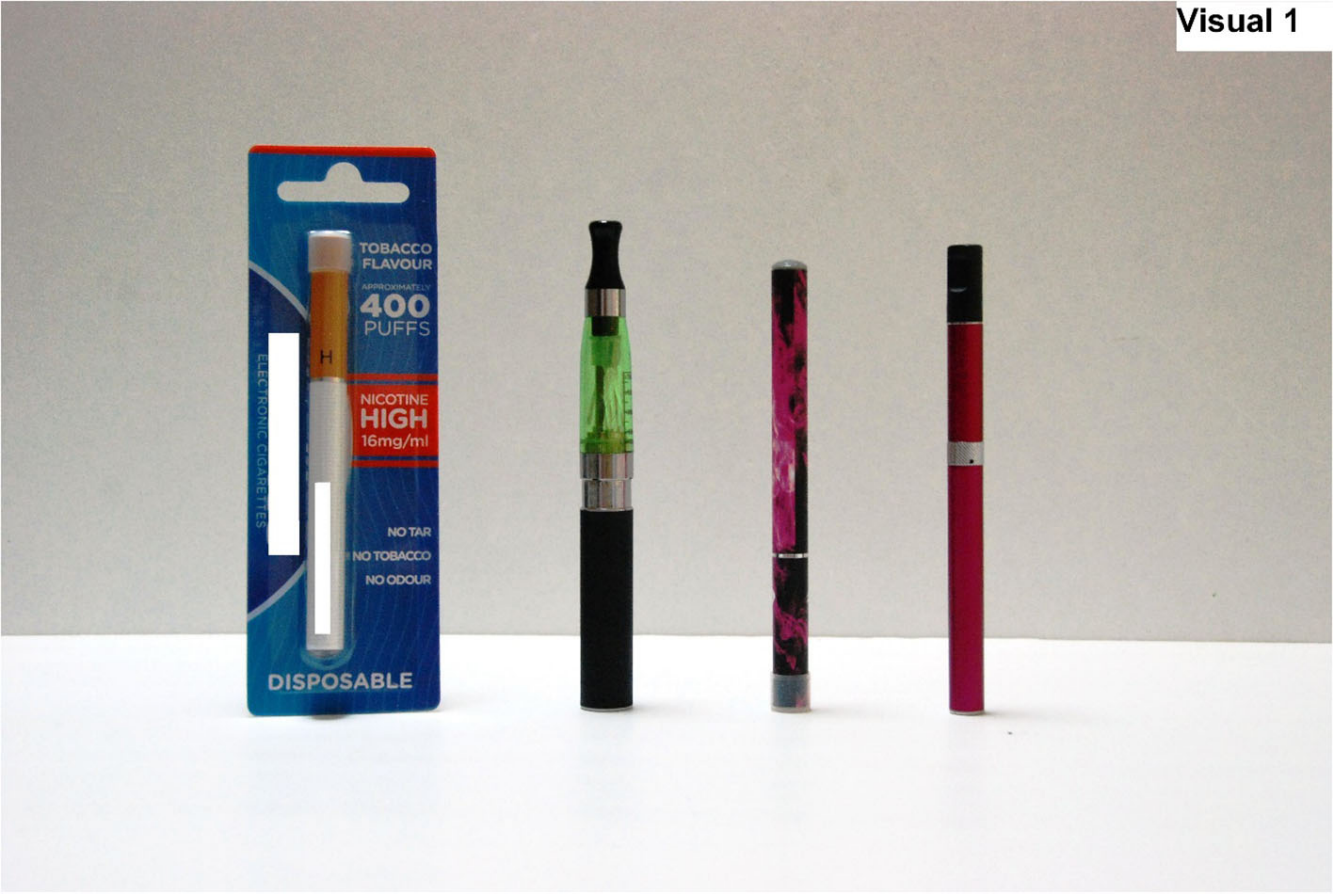
Visual prompt used in the survey to illustrate different styles of e-cigarettes

Subsequent questions on e-cigarettes were asked of all respondents, regardless of whether they had heard of e-cigarettes, by including a description and visual representation of e-cigarettes: ‘E-cigarettes come in different styles. Some look similar to normal cigarettes and have a glowing tip while some look more like pens. Here is a picture of some different styles of e-cigarettes (see Fig. 1). Have you ever seen any of these types of e-cigarettes?’

#### E-cigarette use

One item assessed e-cigarette use: ‘Which of these best describes whether or not you have ever used or tried e-cigarettes?’ Response options were ‘I have never used e-cigarettes’, ‘I have only ever tried e-cigarettes once or twice’, ‘I have used e-cigarettes in the past, but I never use them now’, ‘I occasionally use e-cigarettes (less than once a month)’, ‘I use e-cigarettes at least once a month’, and ‘I use e-cigarettes at least once a week’.

#### Awareness of e-cigarette promotion

Awareness of e-cigarette promotion was assessed via nine items and included TV, radio, newspapers/magazines, posters/billboards, point-of-sale display, social media, sports/games sponsorship, special price offers, and famous people pictured with e-cigarettes. For each type of promotion, participants were presented with a showcard and asked; ‘For each one can you tell me if you have seen anything like this in the last month?’ with response options of ‘Yes’, ‘No’, ‘Don’t know’.

#### E-cigarette brand awareness

**Fig. 2:**
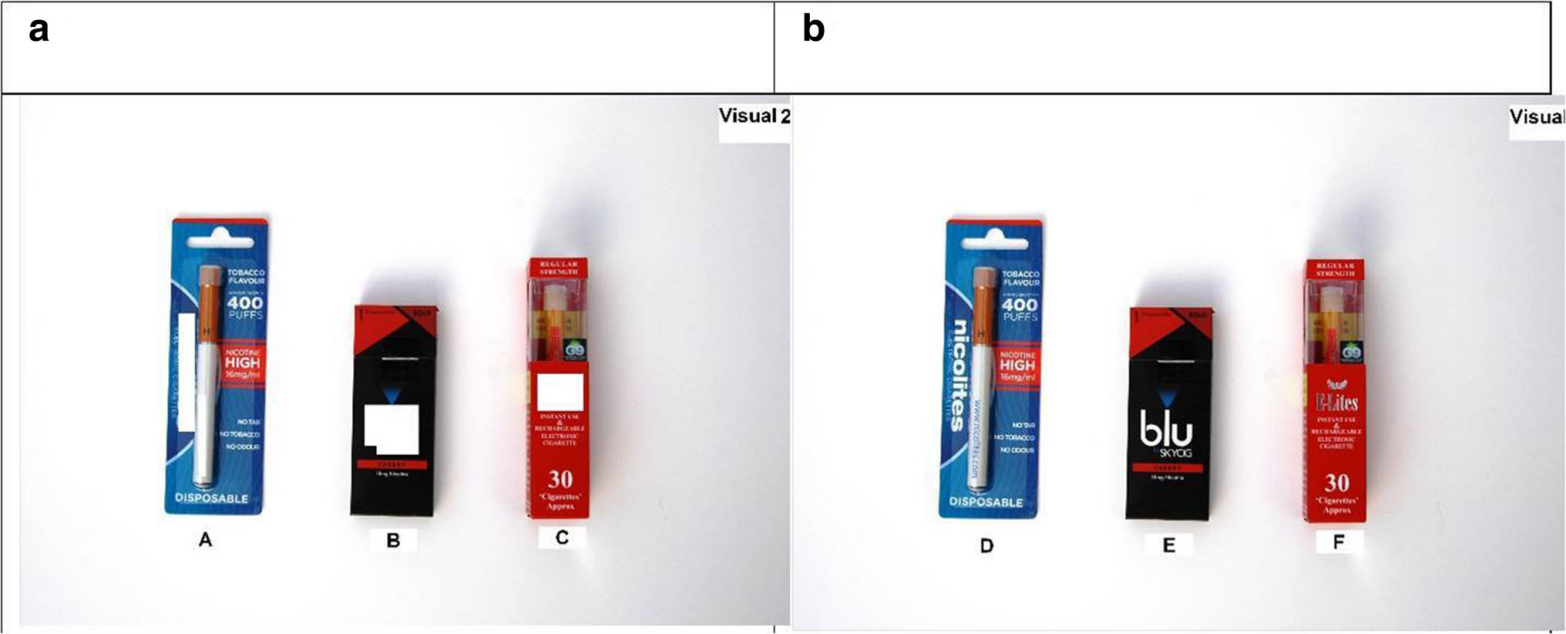
Visual prompt of masked (**a**) and unmasked (**b**) e-cigarette packs to assess e-cigarette brand awareness

Brand awareness was assessed via three items. Brand recall was assessed by asking participants to name brands of e-cigarettes that they had heard of. No prompts were given and a maximum of six brands were recorded. Brand identification was assessed by showing a visual prompt with three brands of e-cigarettes with the brand name masked out (Fig. 2a) and asking them to name each brand. Brand recognition was assessed by showing a visual of the same three brands, but without the brand name masked out, and asking if they had seen each before (Yes/No/Not sure) (Fig. 2b).

#### E-cigarette flavour awareness

Participants were asked: ‘Do you think that e-cigarettes all taste the same or do you think they come in different flavours?’ with response options ‘They come in different flavours’, ‘All taste the same’ and ‘Don’t know’. Those who answered ‘They come in different flavours’ were then asked: ‘Can you tell me any different flavours that you’ve heard of for e-cigarettes’, with a maximum of six flavours recorded.

#### Perceptions of product harm

To provide a general measure of perception of harm, participants were asked ‘Tell me, overall, what you think about people using e-cigarettes’. Participants were then asked to rate how harmful, if at all, four different flavours (tobacco, cherry, candy floss, coffee) would be to the health of the person using it. Responses for all items were provided on a five-point sematic scale ranging from ‘Not at all harmful to health (1)’ to ‘Very harmful to health (5)’.

#### Perceived user image for e-cigarette flavours

Participants were asked to rate how likely or unlikely it would be for ‘an adult who is trying to give up smoking’ to use or try e-cigarettes with different flavours (tobacco, cherry, candy floss and coffee). The same question was also asked for ‘someone their age who has never smoked’. Responses ranged from ‘Very likely (1)’ to ‘Very unlikely (5)’.

### Statistical analysis

Data were analysed using SPSS (version 21). Descriptive data are weighted for age and gender. Paired t-tests were run, on weighted data, to produce mean scores for the following items: (1) perception of harm from a particular flavour of e-cigarettes; (2) perceived likelihood of a particular flavour being used by an adult smoker who is trying to give up (3) perceived likelihood of a particular flavour being tried by someone their age who has never smoked and (4) perceived likelihood of a particular flavour being used by an adult smoker (trying to give up) relative to the perceived likelihood of that same flavour being tried by a never smoker of their age.

As data from all the five-point scales are ordinal, the analysis used non-parametric approaches, initially using the Friedman Test to examine whether responses differed depending on the flavour asked about. Where the Friedman Test detected differences, post hoc tests were conducted using the Wilcoxon signed rank test, a non-parametric procedure suited to paired data. When examining perception of harm from a particular flavour, each flavour was compared against the general measure of harm from e-cigarettes. When examining likelihood of different types of people using each e-cigarette flavour, the tobacco flavour was used as the reference category and compared with each of the other three flavours (cherry, candy floss and coffee). To account for multiple comparisons, a Bonferroni Correction was applied to the critical *p* value, resulting in a *p* value <0.0125 being required for results to reach significance. All descriptive data, including the paired means, are based on weighted data. All non-parametric tests were run on unweighted data. Significance levels quoted are from unweighted non-parametric tests.

## Results

### Sample

A total of 1205 interviews were completed. Excluding cases that were missing for smoking status (*n* = 30), 80 % (*n* = 934) were never smokers (see Table 1).

**Table 1:** Sample profile of survey respondents, UK, 2014

	Unweighted		Weighted^a^	
	*n*	%	*n*	%
Sex				
Male	601	50	602	50
Female	604	50	601	50
	1205		1203	
Age				
11	240	20	200	17
12	182	15	200	17
13	203	17	200	17
14	219	18	200	17
15	189	16	200	17
16	172	14	200	17
	1205		1203	
Social grade				
ABC1 (Higher income group)	480	40	478	40
C2DE (Lower income group)	711	59	711	60
	1191		1189	
Smoking status				
Never smoker	948	81	934	80
Ever smoker	226	19	239	20
Regular	60	5	65	6
Occasional	23	2	25	2
Used to smoke	37	3	39	3
Tried smoking	106	9	110	9
	1174		1173	

^a^Data are weighted to standardise by age and gender

#### Awareness of e-cigarettes and prevalence of use

Eighty-five per cent (*n* = 1025) indicated that they had heard of e-cigarettes and 80 % (*n* = 969) had seen e-cigarettes like those shown in the visual prompt (Fig. 1). Prevalence of ever use of e-cigarettes was 12 % (*n* = 141), with experimentation increasing with age (*p* < 0.001), e.g. 3 % (*n* = 5) of 11 year olds, 17 % (*n* = 33) of 14 year olds and 26 % (*n* = 52) of 16 year olds had tried e-cigarettes. While the majority (83 %, *n* = 117) of those who had tried e-cigarettes were ever smokers, 17 % (*n* = 24) were never smokers. Only 2 % (*n* = 21) used e-cigarettes at least monthly. This occurred among ever smokers where prevalence of monthly e-cigarette use was 9 % (*n* = 21). No regular use was identified in those who had never tried smoking.

#### Brand awareness

Brand awareness was low, with most (84 %, *n* = 1004) unable to recall (unaided) any e-cigarette brands. Sixteen percent (*n* = 189) were able to name one brand of e-cigarettes, while less than 1 % (*n* = 9) could name two. The brands with the highest recall were E-lites (8 %, *n* = 100), Nicolites (2 %, *n* = 22) and Blu/Skycig (2 %, *n* = 27).

For packs with the brand name masked (Fig. 2a) only 1 % (*n* = 7) correctly identified Nicolites, fewer than 1 % (*n* = 1) identified E-lites while none identified Blu. For packs with the brand name visible (Fig. 2b) approximately a third indicated having seen Nicolites (33 % *n* = 399) and E-lites (31 %, *n* = 375) while almost a fifth (17 %, *n* = 210) recognised Blu.

#### Awareness of e-cigarette promotion

**Fig. 3:**
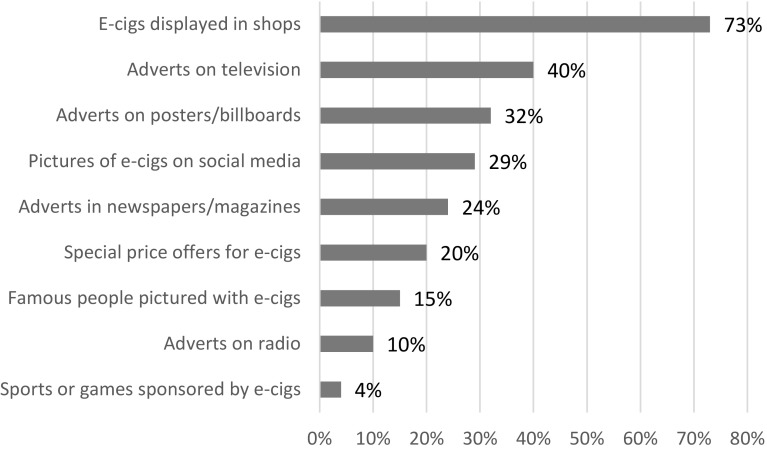
Awareness of e-cigarette marketing among UK adolescents, 2014

Most (82 %, *n* = 990) were aware of at least one type of e-cigarette promotion, with an average of 2.47 channels mentioned (SD = 1.93). The most common channel (Fig. 3) was ‘e-cigarettes being displayed in shops’ (73 %, *n* = 870) followed by ‘adverts on television’ (40 %, *n* = 478), ‘adverts on posters/billboards’ (32 %, *n* = 388) and ‘pictures of e-cigarettes on social media’ (29 %, *n* = 351). Fewer than a quarter were aware of e-cigarette promotion in each of the remaining channels.

#### Awareness of flavours

More than two-thirds (69 %, *n* = 828) were aware that e-cigarettes are available in different flavours. Over half (53 %, *n* = 638) could name at least one e-cigarette flavour with an average of 1.64 flavours mentioned (SD = 1.96). The most frequently mentioned flavours were fruit (45 %, *n* = 542), sweets (18 %, *n* = 218), drinks (17 %, *n* = 201) and tobacco/nicotine (10 %, *n* = 126).

#### Perceptions of flavours

E-cigarettes were, in general, perceived as being harmful (*M* = 3.54, SD = 1.19) with a mean score above the midpoint of the scale. Perceptions of harm from the different flavours ranged from a mean of 3.00 (SD = 1.35) for candy floss flavour to 3.06 (SD = 1.29) for cherry, 3.47 (SD = 1.22) for coffee and 3.99 (SD = 1.14) for tobacco flavour.

Perceptions of harm differed depending on the flavour, *χ*^2^ (4) = 851.59, *p* < 0.001. Post hoc analysis showed that, when compared against perceptions of harm of e-cigarettes in general, tobacco flavour e-cigarettes were perceived as being more harmful (*p* < 0.001) while cherry and candy floss flavours were each perceived as less harmful (*p* < 0.001) (Table 2a). Coffee flavour e-cigarettes were perceived as having the same level of harm as e-cigarettes in general.

**Table 2:** Paired comparison tests for perceptions of harm and user image for different e-cigarette flavours, among UK adolescents, 2014

(a)	How harmful, if at all, do you think …. Would be to the health of the person using it? Not at all harmful (1)/very harmful (5)				(b)	How likely or unlikely do you think it is that AN ADULT SMOKER, WHO IS TRYING TO GIVE UP, would use …. Flavoured e-cigarettes?				How likely or unlikely do you think it is that SOMEONE YOUR AGE, WHO HAS NEVER SMOKED, would use …. Flavoured e-cigarettes?			
	*N*	Mean^a^	SD	*p* value*	Very likely (1)/very unlikely (5)	*N*	Mean^a^	SD	*p* value*	*N*	Mean^a^	SD	*p* value*
E-cig generic	1086	3.53	1.19		Tobacco	1107	2.47	1.47		1121	3.74	1.39	
v				<0.001	v				<0.001				<0.001
Tobacco		4	1.14		Cherry		2.87	1.25			2.75	1.4	
E-cig generic	1088	3.53	1.19		Tobacco	1108	2.46	1.46		1122	3.75	1.39	
v				<0.001	v				<0.001				<0.001
Cherry		3.08	1.29		Candy floss		3.11	1.35			2.67	1.45	
E-cig generic	1091	3.53	1.19		Tobacco	1103	2.47	1.47		1112	3.74	1.39	
v				<0.001	v				<0.001				<0.01
Candy floss		3.02	1.35		Coffee		2.8	1.29			3.64	1.3	
E-cig generic	1073	3.53	1.19										
v				0.234									
Coffee		3.48	1.22										

* Wilcoxon signed rank test for significant differences, with a Bonferroni correction applied resulting in significance level set at *p* < 0.0125

^a^Means from paired *t* tests

An adult smoker, trying to give up smoking, was considered most likely to use a tobacco flavour e-cigarette (*M* = 2.46, SD = 1.47), somewhat likely to use a cherry (*M* = 2.86, SD = 1.24) or coffee (*M* = 2.80, SD = 1.30) flavour e-cigarette, but unlikely to use a candy floss (*M* = 3.10, SD = 1.35) flavour e-cigarette. For a never smoker their own age, they were considered most likely to try candy floss (*M* = 2.65, SD = 1.44) or cherry flavour (*M* = 2.73, SD = 1.40) e-cigarettes but unlikely to try tobacco (*M* = 3.74, SD = 1.39) or coffee (*M* = 3.64, SD = 1.30) flavour e-cigarettes.

Perceptions of likelihood of an adult smoker using each differed depending on the flavour, *χ*^2^ (3) = 153.9, *p* < 0.001 as did perceptions of likelihood of a never smoker of their age *χ*^2^ (3) = 879.01, *p* < 0.001. Post hoc analysis showed that, when compared with tobacco flavour e-cigarettes, adult smokers who were trying to give up smoking were perceived to be less likely to use cherry, candy floss or coffee flavours (*p* < 0.001). Conversely, a never smoker of their age was perceived to be more likely to try cherry (*p* < 0.001), candy floss (*p* < 0.001) or coffee flavour (*p* < 0.01) than a tobacco flavour e-cigarette (Table 2b).

Comparisons of the likelihood of each flavour being used/tried by an adult smoker compared with a never smoker of their age (Table 3a) showed that they perceived that an adult smoker would be more likely than a never smoker of their age to use tobacco (*p* < 0.001) and coffee (*p* < 0.001) flavours whereas a never smoker of their age was perceived to be more likely than an adult smoker to try candy floss (*p* < 0.001) and cherry (*p* < 0.01) flavours.

**Table 3:** Paired comparison tests for perceptions of e-cigarette user image among UK adolescents, 2014

How likely or unlikely do you think it is that adult smoker, trying to give up, v someone your age who has never smoked, would use each flavour of e-cigarettes?									
	(a) Total sample			(b) Never smokers			(c) Ever smokers		
	Mean^a^	SD	*p* value*	Mean^a^	SD	*p* value*	Mean^a^	SD	*p* value*
Very likely (1)/very unlikely (5)									
Tobacco			*N* = 1086			*N* = 833			*N* = 225
Adult	2.46	1.46		2.52	1.48		2.21	1.38	
v			<0.001			<0.001			<0.001
Own age	3.75	1.39		3.84	1.34		3.38	1.47	
Cherry			*N* = 1088			*N* = 830			*N* = 229
Adult	2.86	1.24		2.85	1.24		2.86	1.25	
v			<0.01			0.64			<0.001
Own age	2.71	1.38		2.82	1.4		2.23	1.18	
Candy floss			*N* = 1091			*N* = 835			*N* = 229
Adult	3.1	1.35		3.08	1.35		3.15	1.37	
v			<0.001			<0.001			<0.001
Own age	2.63	1.44		2.75	1.46		2.12	1.19	
Coffee			*N* = 1073			*N* = 821			*N* = 224
Adult	2.79	1.29		2.82	1.29		2.66	1.28	
v			<0.001			<0.001			<0.001
Own age	3.63	1.3		3.72	1.28		3.24	1.31	

* Wilcoxon signed rank test for significant differences, with a Bonferroni correction applied resulting in significance level set at *p* < 0.0125

^a^Means from paired *t* tests

Results were consistent when examined by smoking status (Table 3b, c), except that never smokers considered it equally likely that cherry flavour would be used by an adult smoker or a never smoker their age.

## Discussion

This UK study adds to the literature on e-cigarettes in three ways: it confirms existing data on usage (Bauld et al. 2015); it provides new data on response to promotion and branding; and it gives a first look at reactions to different flavourings. Prevalence of e-cigarette use, at 12 %, is consistent with other studies conducted in the UK in the same 12-month period, including national surveys in Scotland (ISD Scotland 2014) and Wales (Moore et al. 2015). As with these other surveys, only a small proportion of never smokers reported e-cigarette use. No regular use among never smokers was identified here or in the Scotland study, and only a tiny proportion (0.3 %) was reported in the Wales study.

The vast majority of our sample had heard of e-cigarettes and over two-thirds knew that they came in different flavours. There was high awareness of e-cigarette promotion, with most participants aware of at least one type of promotion. However, awareness of e-cigarette branding—a key promotional driver of consumption—was very low. Unlike in the US, where consolidation of the market has created a small number of brand leaders (Giovenco et al. 2014), the UK market remains fragmented. However, US-style rationalisation is expected following tobacco company involvement in the market (Hegarty 2015).

Participants were asked about the absolute harm of e-cigarettes, rather than harm relative to tobacco cigarettes. E-cigarettes were generally seen as harmful and not intended for young people, although these perceptions were influenced by flavour descriptors. For instance, tobacco-flavouring increased harm perceptions, suggesting that awareness of the hazards of tobacco is having an effect. In contrast, fruit and sweet flavours decreased perceptions of harm.

This is the first study to explore adolescents’ awareness of different types of e-cigarette promotion and the influence of flavour descriptors on beliefs of product harm and user image. It benefits from a national sample of UK adolescents. There are a number of study limitations. The cross-sectional nature of the study does not enable causal associations to be explored between awareness of marketing and product-related beliefs or e-cigarette use. While the survey explored awareness of nine different types of e-cigarette promotions, it did not cover all types of promotion. The study also focused on four examples of flavour descriptors: tobacco, cherry, candy floss and coffee. The findings may not apply to other flavours in the same category, for example, an alternative fruit or sweet flavour may not be perceived to be less harmful than a tobacco flavoured e-cigarette.

While the findings suggest that non-smokers are not currently being drawn into using e-cigarettes, there is a need to monitor the situation over time as both the market and regulatory environment develops and changes. Pending regulation in many jurisdictions will significantly restrict e-cigarette marketing. In areas with fewer restrictions, e-cigarette manufacturers may utilise marketing avenues such as free gifts and trials, brand stretching, direct promotional mail, competitions, novel and innovative packaging and product design, and future developments in digital channels to communicate with potential consumers. Further research is needed to monitor e-cigarette marketing strategies, along with adolescents’ exposure to, and involvement with them, and any associated influence on e-cigarette trial and regular use.

The influence of perceptions of product harm and flavours should also be further examined. For adult smokers, flavours can play a useful role in quitting by increasing the appeal of e-cigarettes and helping migration away from tobacco (Farsalinos et al. 2013). In this survey, young people perceived that a never smoker of their age may be more likely to try a candy floss-flavoured e-cigarette than an adult smoker. This highlights the fact that cues like flavour descriptors not only suggest product attributes such as harm, but also other constructs such as user image. While previous studies have suggested little interest among teenagers in trying flavoured e-cigarettes (Shiffman et al. 2015; Pepper et al. 2013), whether user image perceptions makes certain flavours of e-cigarettes attractive to young never smokers, or motivates experimentation, requires further exploration. Marketing literature shows that user image of a product provides a stereotypical view of the generalised user and can shape consumer perceptions and peer acceptance of a product. There is evidence that a cognitive match between an individual’s self-concept and the user image of a product (self-image congruence) (Sirgy 1982) can influence purchase and consumption (Hosany and Martn 2012).

Young people would benefit from clear and consistent information about e-cigarette products and product harm, including their relative harm compared with tobacco cigarettes. E-cigarette marketing is reaching a broad audience and different flavour categories may blur the message about the intended user. These concerns help explain policymakers’ plans to place restrictions on e-cigarette marketing. E-cigarette advertising is permitted in the UK under the Committees for Advertising Practise (CAP) rules which state that advertisements must not encourage non-smokers and non-nicotine users to use e-cigarettes and must not appeal to anyone under 18 (Committees of Advertising Practice 2014). In 2016, however, the European Union’s Tobacco Products Directive (TPD) will override EU member states’ current domestic arrangements and impose a ban on e-cigarette advertising, promotion and sponsorship, similar to current restrictions for tobacco products (European Parliament and the Council of the European Union 2014). Furthermore, the TPD allows member states to regulate the availability of flavours. Wills et al. (2015) have noted that attention should be given to e-cigarette marketing and the perceived attractiveness of e-cigarettes because of flavourings. This study reinforces their point.
